# Assessing Rat Forelimb and Hindlimb Motor Unit Connectivity as Objective and Robust Biomarkers of Spinal Motor Neuron Function

**DOI:** 10.1038/s41598-019-53235-w

**Published:** 2019-11-13

**Authors:** Markus E. Harrigan, Angela R. Filous, Andrew P. Tosolini, Renee Morris, Jan M. Schwab, W. David Arnold

**Affiliations:** 1 Department of Neurology, Spinal Cord Injury Medicine (Paraplegiology), The Ohio State University, Wexner Medical Center, Columbus, OH USA; 2 Department of Neuromuscular Diseases, Institute of Neurology, University College London, London, WC1N 3BG UK; 3 Translational Neuroscience Facility, School of Medical Sciences, The University of New South Wales, Sydney, NSW 2052 Australia; 4 Belford Center for Spinal Cord Injury, The Ohio State University, Wexner Medical Center, Columbus, OH USA; 5 Department of Neurology, Neuromuscular Division, The Ohio State University, Wexner Medical Center, Columbus, OH USA; 6 Department of Physical Medicine and Rehabilitation, The Ohio State University, Wexner Medical Center, Columbus, OH USA; 7 Department of Neuroscience, The Ohio State University, Wexner Medical Center, Columbus, OH USA; 8 Department of Physiology and Cell Biology, The Ohio State University, Wexner Medical Center, Columbus, OH USA

**Keywords:** Electromyography - EMG, Diseases of the nervous system, Peripheral nervous system, Motor neuron disease, Spinal cord diseases

## Abstract

Sensitive and objective biomarkers of neuronal injury, degeneration, and regeneration can help facilitate translation of experimental findings into clinical testing. Whereas measures of upper motor neuron connectivity have been readily established, functional assessments of lower motor neuron (LMN) innervation of forelimb muscles are lacking. Compound muscle action potential (CMAP) and motor unit (MU) number estimation (MUNE) are well-established methods that allow longitudinal MU integrity monitoring in patients. In analogy we refined CMAP and MUNE methods for assessing spinal MU input in the rat forelimb and hindlimb. Repeated CMAP and MUNE recordings are robust (coefficients of variability: 4.5–11.3%), and MUNE measurements from forelimb wrist flexor muscles (415 ± 8 [SEM]) align with back-traced anatomical LMN counts (336 ± 16 [SEM]). For disease validation, cross-sectional blinded electrophysiological and muscle contractility measurements were obtained in a cohort of G93A SOD1 mutant overexpressing rats and compared with controls. Longitudinal assessment of mutant animals demonstrated progressive motor unit decline in the hindlimb to a greater extent than the forelimb. Hindlimb CMAP and MUNE demonstrated strong correlations with plantarflexion muscle contractility. Cross-species assessment of upper/fore- limb and lower/hind- limb motor units using objective electrophysiological CMAP and MUNE values as biomarkers will guide and improve bi-directional translation.

## Introduction

The motor unit (MU) is composed of a single spinal motor neuron and the muscle fibres it innervates. Activation of the MU pool generates muscle contractions as the final execution of voluntary movement. A number of electrophysiological strategies can be used to assess and track the integrity of the MU pool *in vivo*. Compound muscle action potential (CMAP) represents the summated depolarization of all muscle fibres in a particular muscle or group of muscles following peripheral nerve stimulation. CMAP provides an assessment of the total excitability or electrophysiological output from a particular muscle or muscle group and is sensitive to a variety of neuromuscular disease states, including amyotrophic lateral sclerosis (ALS)^1,2^, spinal muscular atrophy (SMA)^3,4^, peripheral neuropathy, and critical illness myopathy^5,6^. One limitation of CMAP measures is the fact that collateral sprouting can result in preserved CMAP amplitude and area despite MU loss. To address this limitation, the CMAP technique has been modified to assess MU number and size.

The incremental MU number estimation (MUNE) technique was first reported in the early 1970s in the extensor digitorum brevis muscle in humans as a means to track the number of motor neurons functionally connected to a particular muscle^7^. In this technique, sub-maximal stimulations of a peripheral nerve are used to obtain an average single MU potential (SMUP) amplitude representing the number of muscle fibres innervated by one single motor neuron. MUNE can sensitively identify and track motor unit loss and is thus able to identify MU dysfunction prior to other measures such as CMAP amplitude or area^8,9^. This is most strikingly demonstrated in ALS patients, where MUNE has emerged as one of the most sensitive biomarkers of disease onset, progression, and prognosis^10,11^. Multiple variations of MUNE have been developed and extensively applied to monitor MU function in neurodegeneration, neurotrauma, and the normal process of aging^12–15^.

In rodent disease models, various labelling, such as with retrograde tracers and adenoviruses, can quantify the number of innervating motor neurons^16–19^. Labelling techniques are limited as terminal readouts and do not interrogate motor neuron functionality. In contrast, MUNE allows for a functional readout of motor neuron connectivity with muscle. MUNE can be applied longitudinally to understand disease course and to test protective or regenerative effects of therapeutic interventions, both in the clinic and in preclinical studies. Despite the powerful nature of repeated MUNE measures and the clinical applicability of the technique to MU pools across most of the human body, application of this technique has been limited to the hindlimb in rodent models^17,20–22^. An objective *in vivo* quantification of the spinal motor neurons supplying the forelimb musculature remains elusive in animal models.

**Figure 1:**
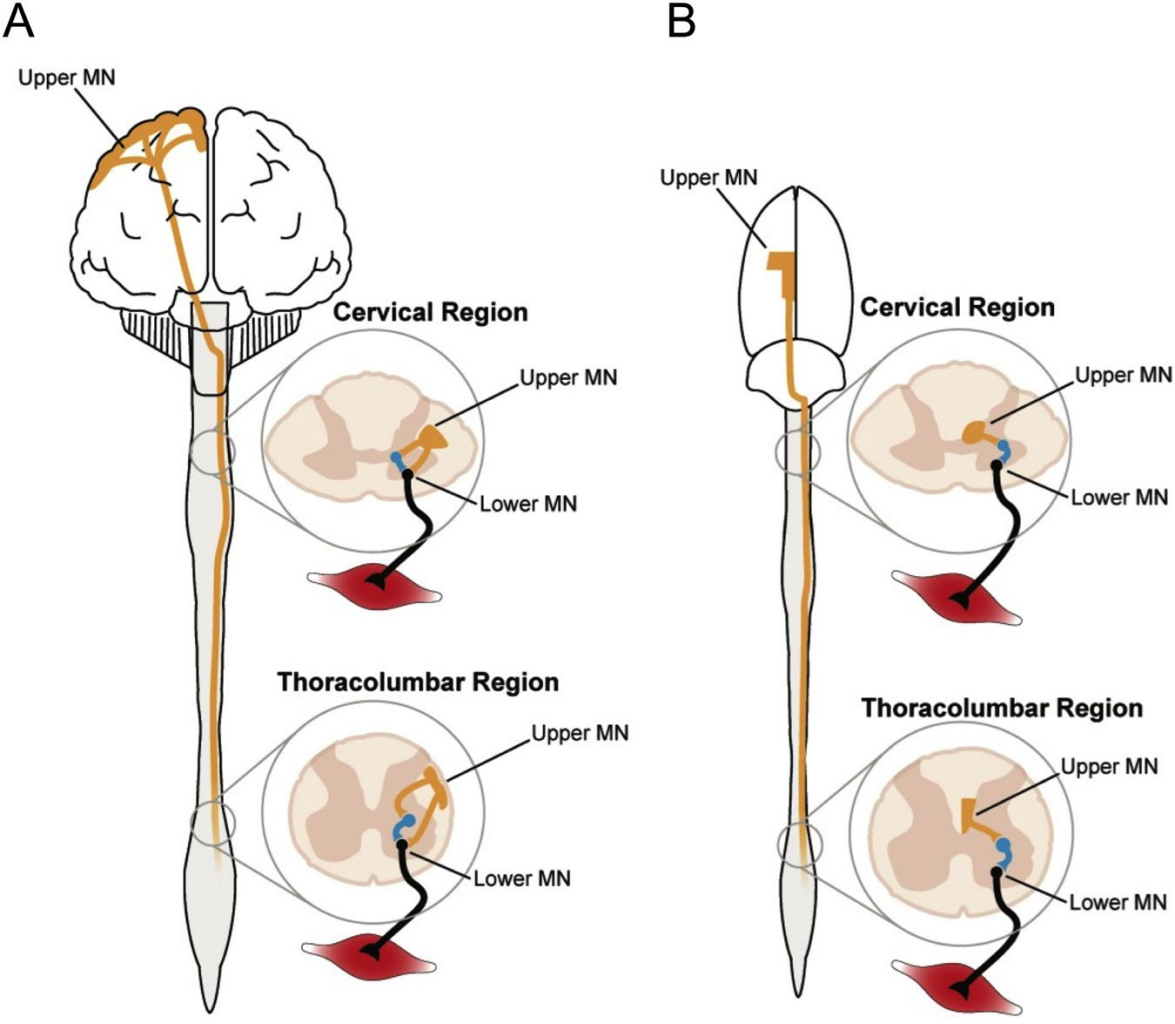
Species-divergent organisation of motor systems. **(A**) The corticospinal tracts (CSTs; Upper motor neuron [UMN]; orange) in the human spinal cord are localised in the dorsolateral (pictured) and ventral compartment of the spinal cord and synapse directly on both interneurons (blue) and spinal motor neurons (Lower motor neuron [LMN]; black). Superior dexterity in non-human primates and humans, relative to rats, correlates with development of the CST^59^. (**B**) Relative to human CSTs, the rat CSTs comprise considerably less fibres, which are also smaller in size. Rat CSTs are located in the lateral, ventral, and deep dorsal (pictured) columns and only synapse directly on interneurons, which in turn synapse on spinal motor neurons^19,24^. Further, rats and humans share the same number of cervical spine segments (8), but an extra segment is present in both the thoracic (T13) and lumbar (L6) regions in the rat. The images in this figure were designed by Timothy Warner and are presented here with his permission.

Objective electrophysiological measures are needed for more effective cross-species translation, as prior work has uncovered pronounced fundamental differences in the anatomical and functional characteristics of the motor systems of primates versus rodents (Fig. 1)^23,24^. The ability to factor in neurobiological differences has been limited and constitutes an obstacle for the translation of rodent models to patients. Recently, a technique measuring ulnar nerve excitability was established in the rat forelimb, but these recordings assay ion channel function and membrane potential and do not provide a quantitative readout of motor unit pool function^25^. MUNE surmounts these barriers as an objective electrophysiological readout of LMN function that can be utilised to assess functional motor unit connectivity as a biomarker in experimental spinal cord and motor neuron disease models.

In order to expand the applicability and use of measures of MU connectivity in clinically relevant rodent models, we investigated three aims in three sets of experiments: First, we aimed to establish feasibility and robustness of CMAP and MUNE recording techniques in the wild type rat forelimb and hindlimb through repeated measurements to determine mean values and test-retest reliability. Then, we aimed to determine whether our electrophysiological estimates reasonably align with spinal cord anatomy by comparing electrophysiologically-calculated MU numbers with motor neuron counts determined by retrograde anatomical tracer analysis. Lastly, we sought to validate CMAP and MUNE as sensitive objective biomarkers of longitudinal motor unit degeneration and dysfunction by obtaining repeated recordings from SOD1 transgenic rats, which model ALS.

## Results

### Replicability and robustness of electrophysiological motor unit recordings from the rat forelimb and hindlimb

**Figure 2:**
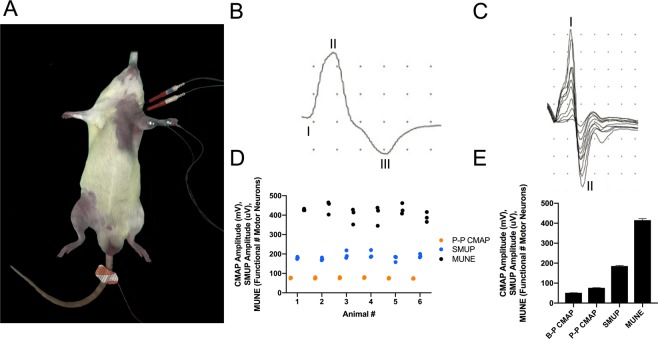
Functional motor units innervating forelimb muscles were assessed by motor unit number estimation *in vivo*. (**A**) Electrophysiological setup to record compound muscle action potential (CMAP) and incremental single motor unit potentials (SMUPs) in the left ventromedial forearm (forelimb), which houses the wrist flexor muscles (flexor carpi radialis m., flexor digitorum profundus m., palmaris longus m., flexor digitorum superficialis m.). The red needle electrodes are stimulating electrodes, which straddle the brachial plexus. The active recording electrode (E1) is placed on the left ventromedial forearm and the reference recording electrode (E2) overlies the left wrist while a ground electrode is in contact with the tail. Anaesthesia is induced and maintained with isoflurane, and normal body temperature is preserved with a heating pad (see Methods section for more details). (**B**) Representative CMAP response from the ventromedial forearm region. Baseline-to-Peak (B-P) CMAP amplitude is determined by measuring between the baseline (I) and the negative peak (II) of the waveform, while Peak-to-Peak (P-P) CMAP amplitude is determined by measuring between the negative peak (II) and the positive peak (III). X-axis represents time (ms) and y-axis represents voltage (mV). Sensitivity: 200 mV, 20 mV per division; Duration: 10 ms, 1 ms per division. (**C**) Representative incremental acquisition of ten SMUP waveforms. SMUP amplitude is determined by measuring between negative peak (I) and positive peak (II). X-axis represents time (ms) and y-axis represents voltage (µV). Sensitivity: 2 mV, 200 µV per division; Duration: 10 ms, 1 ms per division. (**E**) Baseline values of B-P CMAP (51.3 ± 0.7), P-P CMAP (76.5 ± 0.7), SMUP (185.4 ± 3.6) and motor unit number estimation (MUNE; 415 ± 8) obtained from recordings in naïve rats. Data presented as mean ± SEM and n = 18 for each value. (**D**) Test-retest of values involved in MUNE calculation: P-P CMAP (), SMUP (), and MUNE values (●) obtained from the wrist flexors of naïve rats; n = 6 rats, with three tests per animal. Low test-retest variation is evident by mean coefficient of variation values (P-P CMAP: 4.5%; SMUP: 8.4%; MUNE: 9.4%).

In the forelimb, we recorded from the ventromedial forearm (Fig. 2A), which overlies the wrist flexor muscles (flexor carpi radialis m., palmaris longus m., flexor digitorum profundus m., flexor digitorum superficialis m.). Forelimb CMAP (Fig. 2B) and SMUP (Fig. 2C) responses were readily attainable and were distinct in appearance as compared with hindlimb responses (see below). The mean baseline-to-peak CMAP (51.3 ± 0.7), peak-to-peak CMAP (76.5 ± 0.7), and SMUP (185.4 ± 3.6) values across all six naïve rats are displayed in Fig. 2E. In three separate test-retest experiments, the mean MUNE value of the wrist flexors for each animal was 427 ± 3, 442 ± 20, 398 ± 23, 402 ± 29, 431 ± 16, and 389 ± 15, while the mean MUNE value of the wrist flexor muscles across all animals was 415 ± 8 (Fig. 2D,E). Forelimb assessments demonstrated consistently low test-retest variation (Fig. 2D), as assessed by the mean coefficient of variation (CMAP: 4.5%; SMUP: 8.4%; MUNE: 9.4%).

**Figure 3:**
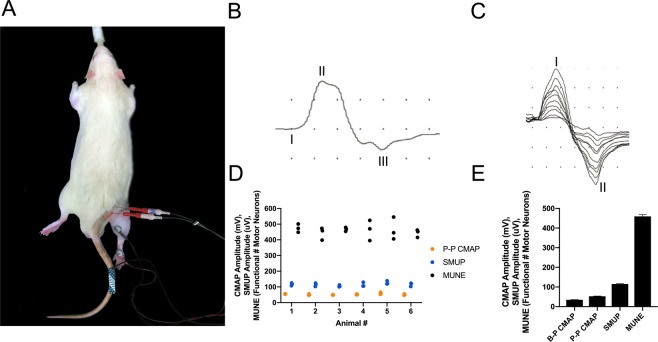
Functional motor units innervating hindlimb muscles were assessed by MUNE *in vivo*. (**A**) Electrophysiological setup to record compound muscle action potential (CMAP) and incremental single motor unit potentials (SMUPs) from the triceps surae muscle region, which comprises the lateral gastrocnemius, medial gastrocnemius, and soleus mm. The red needle electrodes are stimulating electrodes, which straddle the proximal sciatic nerve. The active recording electrode (E1) and reference recording electrode (E2) are placed over the bulk of the right triceps surae and the right heel, respectively, while the ground electrode is on the tail. Anaesthesia is induced and maintained with isoflurane, and normal body temperature is preserved with a heating pad (see Methods section for more details). (**B)** Representative CMAP response from the rat triceps surae region. Baseline-to-Peak (B-P) CMAP amplitude is determined by measuring between baseline (I) and the negative peak (II) of the waveform, while Peak-to-Peak (P-P) CMAP amplitude is determined by measuring between the negative peak (II) and the positive peak (III). Body temperature is maintained at 37 °C to avoid fluctuations in CMAP or SMUP responses. X-axis represents time (ms) and y-axis represents voltage (mV). Sensitivity: 200 mV, 20 mV per division; Duration: 10 ms, 1 ms per division. (**C)** Representative incremental acquisition of ten SMUP waveforms. SMUP amplitude is determined by measuring between negative peak (I) and positive peak (II). X-axis represents time (ms) and y-axis represents voltage (µV). Sensitivity: 2 mV, 200 µV per division; Duration: 10 ms, 1 ms per division. (**E**) Baseline values of B-P CMAP (34.7 ± 0.7), P-P CMAP (52.7 ± 1.1), SMUP (115.3 ± 2.6), and motor unit number estimation (MUNE; 459 ± 9) values obtained from recordings in naïve rats. Data presented as mean ± SEM and n = 18 for each value. (**D**) Test-retest of values involved in MUNE calculation: P-P CMAP (), SMUP (), and MUNE values (●) obtained from the triceps surae of naïve rats; n = 6 rats, with three tests per animal. Low mean coefficient of variation values indicates minimal test-retest variation (P-P CMAP: 8.1%; SMUP: 10.9%; MUNE: 11.3%).

To assess MU connectivity of the hindlimb, we recorded from the region overlying the triceps surae muscle group (Fig. 3A; medial gastrocnemius m., lateral gastrocnemius m., soleus m.). The mean baseline-to-peak CMAP, peak-to-peak CMAP (Fig. 3B), and SMUP (Fig. 3C) values across all six naïve rats were 34.7 ± 0.7, 52.7 ± 1.1, and 115.3 ± 2.6, respectively (Fig. 3E). In three separate test-retest experiments, the mean MUNE value of the triceps surae for each animal was 474 ± 15, 443 ± 23, 467 ± 7, 463 ± 37, 465 ± 41 and 443 ± 14, while the mean MUNE value of the triceps surae across all animals was 459 ± 10 (Fig. 3D,E). Hindlimb assessments demonstrated consistently low test-retest variation (Fig. 3D) as assessed by the mean coefficient of variation (CMAP: 8.1%, SMUP: 10.9%, and MUNE: 11.3%).

### Anatomical motor neuron counts by retrograde fluoro-gold labelling (rat wrist flexor muscles)

**Figure 4:**
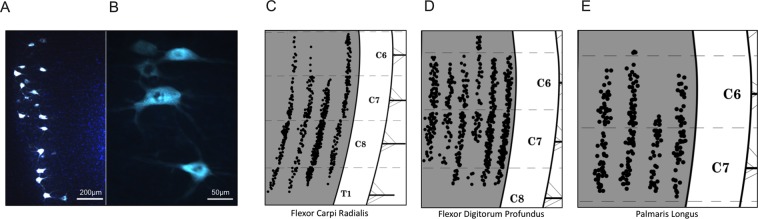
Motor neurons innervating the rat wrist flexor muscles were anatomically detected by intramuscular injection of Fluoro-Gold (FG) and retrograde tracing into the cervical spinal cord. (**A**) A low and (**B**) high magnification representative image of FG-labelled motor neurons (scale bar: 200 µm and 50 µm, respectively). (**C–E**) Schematic spatial representations of the FG-labelled motor neurons after injections in (**C**) flexor carpi radialis, (**D**) flexor digitorum profundus, and (**E**) palmaris longus, reproduced from Tosolini and Morris (2012) with permission from the publisher. Each black dot represents a single FG-labelled motor neuron, and the schematic includes the caudal cervical and first thoracic segment borders, grey/white matter boundaries, and the ventral roots.

Intramuscular injections of Fluoro-Gold (FG) resulted in robust labelling in corresponding motor neurons (Fig. 4A,B). Columns of labelled motor neurons were represented on a diagrammatic schematic for multiple forelimb muscles in Fig. 4 from Tosolini and Morris (2012), which we modified and partly reproduced here for the muscles of interest: flexor carpi radialis m. (FCR; Fig. 4C), flexor digitorum profundus m. (FDP; Fig. 4D) and palmaris longus m. (PL; Fig. 4E). Moreover, these schematics were reanalysed to quantify the total number of labelled motor neurons, the results of which are presented in Table 1. The FCR was injected with FG in four separate animals, resulting in 133, 119, 250, and 158 labelled motor neurons (mean = 165 ± 34). The FDP was injected with FG in six separate animals, resulting in 109, 66, 93, 60, 159, and 163 labelled motor neurons (mean = 108 ± 20). The PL was injected with FG in four separate animals, resulting in 72, 78, 42, and 57 labelled motor neurons (mean = 62 ± 9). For the purposes of comparing the MUNE estimates with the neuroanatomical tracer counts, the summated mean number of FG-labelled motor neurons of all three wrist flexor muscles was 336 ± 16, which is also presented in Table 1.

**Table 1:** Motor neuron pool innervating forelimb muscles: Anatomical and *in vivo* electrophysiological assessment.

**Muscle: Flexor carpi radialis (FCR)**	**# of FluoroGold-labelled motor neurons per animal**
Animal 1	133
Animal 2	119
Animal 3	250
Animal 4	158
Mean	165 ± 34 (SEM)
**Muscle: Flexor digitorum profundus (FDP)**	**# of FluoroGold-labelled motor neurons per animal**
Animal 1	109
Animal 2	66
Animal 3	93
Animal 4	60
Animal 5	159
Animal 6	163
Mean	108 ± 20 (SEM)
**Muscle: Palmaris longus (PL)**	**# of FluoroGold-labelled motor neurons per animal**
Animal 1	72
Animal 2	78
Animal 3	42
Animal 4	57
Mean	62 ± 9 (SEM)
**Summated Anatomical Motor Neuron # Count: FCR + FDP + PL**	**Electrophysiological Motor Neuron # Estimation:**
336 ± 16 (SEM)	415 ± 8 (SEM)

Individual and mean numbers of Fluoro-Gold (FG)-labelled motor neurons ± standard error of the mean (SEM) were determined after FG injections into corresponding forelimb muscles: flexor carpi radialis m. (FCR), flexor digitorum profundus m. (FDP), and palmaris longus m. (PL). These anatomical counts were summated and compared with *in vivo* electrophysiological motor neuron counts for neurobiological validation. The anatomical count (336 ± 16 [SEM]) represents the summated mean number of FG-labelled motor neurons for all assessed wrist flexor muscles (FCR, FDP, PL), while the electrophysiological estimate (415 ± 8 [SEM]) represents the mean calculated MUNE (compound muscle action potential/average single motor unit potential) from ventromedial forearm wrist flexor muscle recordings.

### Baseline electrophysiology and muscle contractility in sod1 mutant and control rats

**Figure 5:**
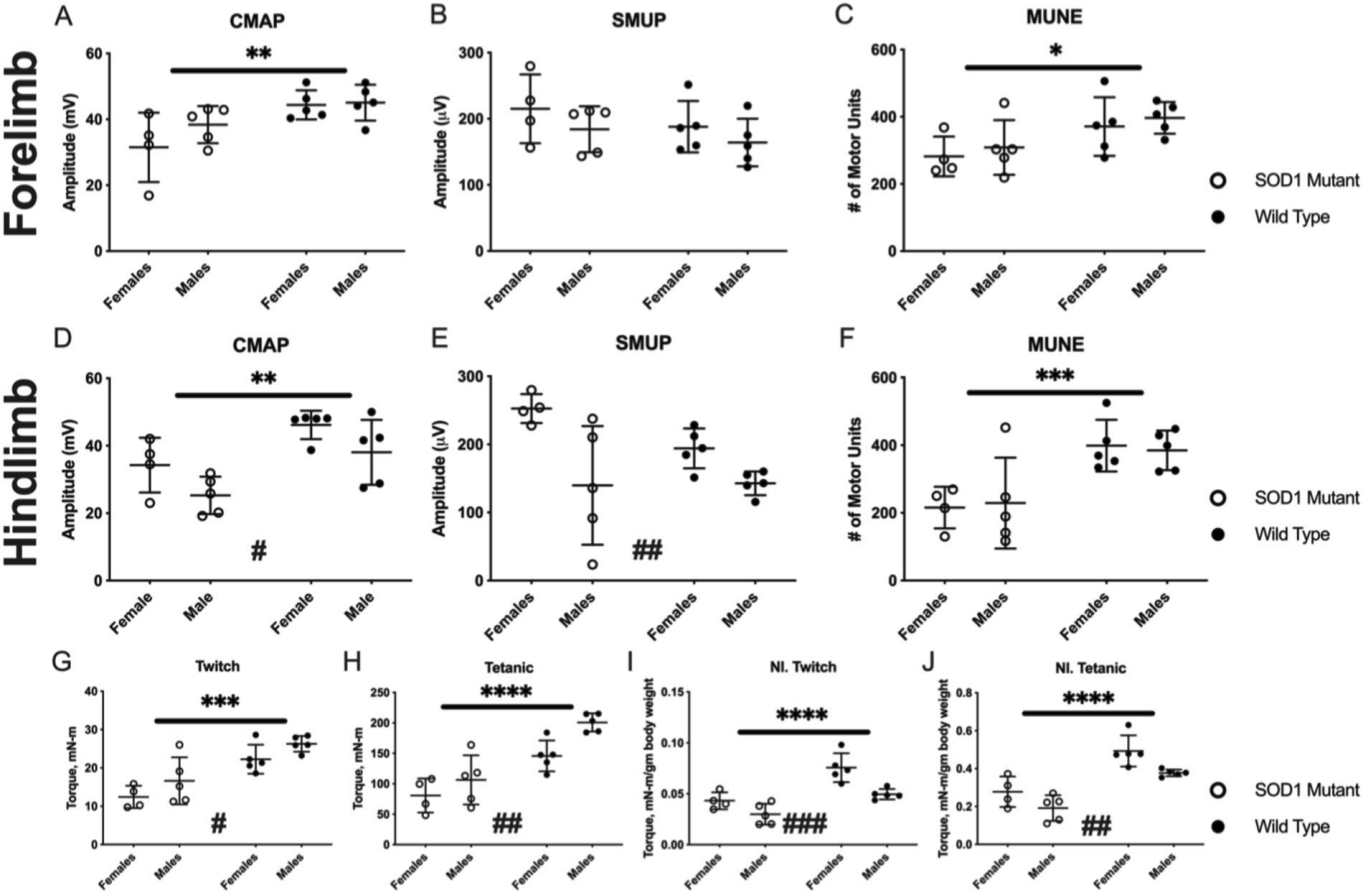
Baseline forelimb (**A–C**) and hindlimb (**D–F**) electrophysiology assessment demonstrated loss of motor unit connectivity in mutant SOD1 rats, while hindlimb muscle contractility (**G–J**) was also reduced in mutant SOD1 rats. (**A**) Forelimb compound muscle action potential (CMAP) demonstrated significant differences in genotype (p = 0.006) but no differences for sex (p = 0.232) or significant interaction (p = 0.323). (**B**) Forelimb single motor unit potential (SMUP) demonstrated no differences for genotype (p = 0.222) or sex (p = 0.160). (**C**) Forelimb motor unit number estimation (MUNE) demonstrated significant differences for genotype (p = 0.017) but no differences for sex (p = 0.438) and no interaction (p = 0.986). (**D**) Hindlimb compound muscle action potential (CMAP) demonstrated significant differences for sex (p = 0.020) and genotype (p = 0.002) but no significant interaction (p = 0.985). (**E**) Hindlimb single motor unit potential (SMUP) demonstrated differences for sex (p = 0.003) but no differences for genotype (p = 0.241) and no interaction (p = 0.195). (**F**) Hindlimb motor unit number estimation (MUNE) demonstrated significant differences for genotype (p < 0.001) but no differences for sex (p = 0.991) and no interaction (p = 0.744). Electrophysiology data presented as mean ± standard deviation and comparisons performed using two-way ANOVA. Mutant SOD1 rats are presented as ○ and wild type control rats as ●. Asterisks indicate statistical differences in genotype: *p < 0.05, **p < 0.01, ***p < 0.001. Number signs indicate statistical differences in sex: ^#^p < 0.05, ^##^p < 0.01. (**G**) Absolute triceps surae (hindlimb) twitch torque demonstrated significant differences for genotype (p < 0.001) and sex (p = 0.046) with no significant interaction (p = 0.962). (**H**) Absolute triceps surae tetanic torque demonstrated differences for genotype (p < 0.001) and sex (p = 0.008) with no significant interaction (p = 0.284). (**I**) Normalised twitch torque demonstrated significant differences for genotype (p < 0.001) and sex (p < 0.001) with no significant interaction (p = 0.188). (**J**) Normalised tetanic torque demonstrated significant differences for genotype (p < 0.001) and sex (p = 0.0048) with no significant interaction (p = 0.635). Hindlimb contractility data presented as mean ± standard deviation and comparisons performed using two-way ANOVA. Mutant SOD1 rats are presented as ○ and wild type control rats as ●. Significant differences for genotype, but not sex, were illustrated. Asterisks indicate differences in genotype: ***p < 0.001, ****p < 0.0001. Number signs indicate statistical differences in sex: ^#^p < 0.05, ^##^p < 0.01, ^###^p < 0.001.

Despite the absence of clinically observable motor weakness in any animals at baseline, electrophysiology and muscle contractility measures identified deficits in mutant SOD1 rats compared to wild type animals. Electrophysiological measurements from forelimb wrist flexor muscles in SOD1 mutant rats demonstrated significant differences compared with measurements in wild type controls (Fig. 5A–C). Both CMAP and MUNE forelimb wrist flexor measurements were significantly reduced in SOD1 rats relative to wild type controls, but there was no difference in SMUP amplitude between genotypes. Similarly, hindlimb triceps surae CMAP and MUNE were reduced in SOD1 rats compared to wild type rats, while SMUP was once again unchanged with respect to genotype (Fig. 5D–F). There were no gender-specific differences in forelimb electrophysiological measures; in the hindlimb, males exhibited lower CMAP values and females displayed higher SMUP values. Compared with control rats, hindlimb plantar flexor muscle contractility was significantly decreased in SOD1 rats for both twitch and tetanic forces when analysed as absolute values (Fig. 5G,H) and when normalised to body mass (Fig. 5I,J). Irrespective of genotype, female rats consistently generated lower absolute forces, but higher normalised forces, compared to males.

### Longitudinal decline of motor unit connectivity and muscle contractility in SOD1 mutant rats

**Figure 6:**
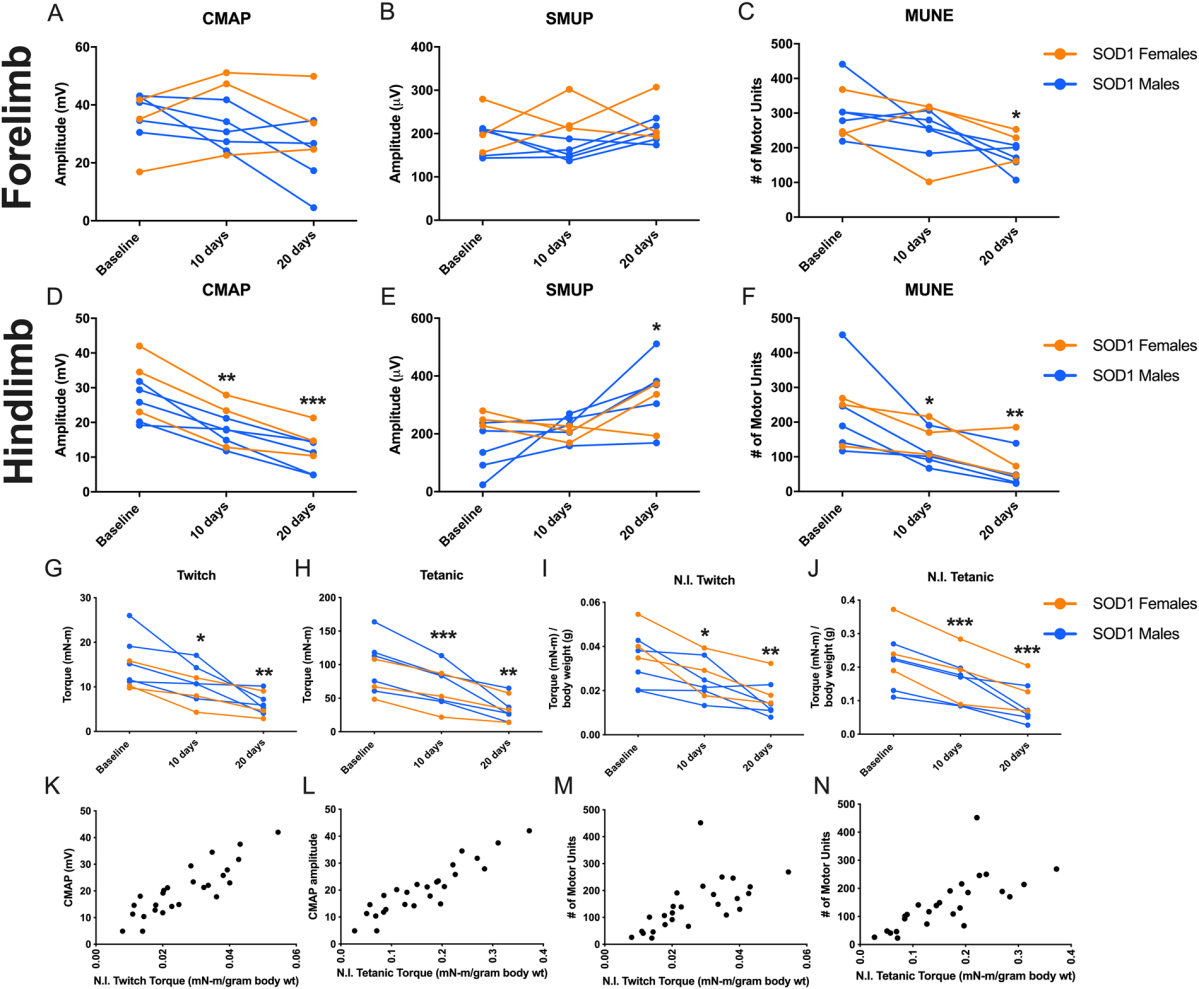
Longitudinal forelimb (**A–C**) and hindlimb (**D–F**) electrophysiology assessment demonstrated rapid loss of motor unit connectivity in mutant SOD1 rats. Additionally, longitudinal plantar flexion muscle contractility assessment (**G–J**) demonstrated rapid loss of torque in mutant SOD1 rats, which was closely correlated with hindlimb electrophysiological measures (**K–N**). There were rapid changes in (**A**) forelimb compound muscle action potential amplitude (p = 0.157), and (**C)** forelimb motor unit number estimation was significantly reduced (p = 0.008), but (**B)** forelimb single motor unit potential amplitude (p = 0.541) was not significantly changed. By contrast, (**D**) hindlimb compound muscle action potential amplitude (p < 0.0001), (**E**) hindlimb single motor unit potential amplitude (p = 0.015), and (**F**) hindlimb motor unit number estimation (p < 0.001) all exhibited significant decline over 20 days after baseline. Mutant SOD1 females (n = 3) are presented as  and mutant SOD1 males (n = 5) as . For electrophysiology data, repeated measure one-way ANOVA with Dunnett’s multiple comparison tests was used to compare assessments at 10 days and 20 days to baseline. Adjusted p values for Dunnett’s multiple comparison tests are illustrated: *p < 0.05, **p < 0.01, ***p < 0.001. Following baseline hindlimb contractility measurements there was rapid loss of (**G**) twitch (p = 0.0004) and (**H**) tetanic (p = 0.0009) contractility. Similarly, when normalised to body mass, (**I**) normalised twitch, (p < 0.0001) and (**J**) normalised tetanic (p < 0.0001) contractility were reduced. Females (n = 3) are shown as  and males (n = 5) as . For muscle contractility data, repeated measure one-way ANOVA with Dunnett’s multiple comparison tests was used to compare assessments at 10 days and 20 days to baseline. Adjusted p values for Dunnett’s multiple comparison tests are illustrated: *<0.05, **<0.01, ***<0.001. Plantarflexion muscle contractility was tightly associated with (**K,L**) compound muscle action potential (CMAP) amplitude (twitch: r = 0.868, p < 0.0001; tetanic: r = 0.911, p < 0.0001) and (**M,N)** motor unit number, as determined by Motor Unit Number Estimation (MUNE), (twitch: r = 0.633, p = 0.0005; tetanic: r = 0.845 p < 0.0001) in mutant male and female SOD1 rats.

We were interested in understanding the rate of decline in forelimb and hindlimb muscles for measures of CMAP, SMUP, and MUNE, as well as the relationship between hindlimb electrophysiological measures and hindlimb plantar flexion contraction torque. Mutant rats were assessed at 10 and 20 days following the baseline measures. During this timeframe, one rat met endpoint criteria for euthanasia and was therefore removed from the study. Forelimb wrist flexor MUNE (Fig. 6C) was significantly reduced at 20 days but not at 10 days, relative to baseline measurements; there were no differences in CMAP and SMUP values (Fig. 6A,B). Hindlimb CMAP and MUNE were reduced at both 10 and 20 days relative to the baseline measurement (Fig. 6D,F). Hindlimb SMUP, an index for motor unit size that is expected to increase with collateral sprouting, was not significantly increased at 10 days but was increased at 20 days following baseline (Fig. 6E**)**. Following baseline assessment, both twitch and tetanic hindlimb muscle contractility demonstrated decline at 10 days relative to baseline when considered as absolute values and when normalised to body mass (Fig. 6G–J). Absolute and normalised twitch and tetanic forces were even more profoundly decreased at 20 days (Fig. 6G–J), a progressive decline that corresponds with the deterioration in hindlimb motor units identified by MUNE (Fig. 6F).

### Association between electrophysiological measures, muscle contractility and motor symptoms

In the hindlimb, CMAP amplitude (Fig. 6K,L) and motor unit number (Fig. 6M,N) were strongly positively correlated with normalised twitch and tetanic torque. There was a moderate correlation between the longitudinal loss of normalised twitch contractilty with longitudinal CMAP decrease (r = 0.375, p = 0.360) and longitudinal MUNE decrease (r = 0.511, p = 0.195) (not shown in Fig. 6). There was also moderate correlation between the longitudinal loss of normalised tetanic contracility with longitudinal CMAP decrease (r = 0.414, p = 0.307) and longitudinal MUNE decrease (r = 0.599, p = 0.117) (not shown in Fig. 6). During the course of our longitudinal studies, we also assessed for signs of motor weakness and identified clinical weakness in 4 of 9 mutant SOD1 rats (2 females and 2 males; “clinically symptomatic”) while the remaining mutant SOD1 rats did not display motor weakness (“clinically pre-symptomatic”). Longitudinal Matsumoto Motor Scores for the rats displaying observable motor weakness are presented in Table 2. We were interested in understanding the magnitude of motor unit loss at symptomatic onset (as defined by a Motor Score of 4) in the clinically symptomatic mutant SOD1 rats. Therefore we compared the forelimb MUNE and hindlimb MUNE results obtained at the timepoint when the Motor Scores reached 4 (Table 2). These results showed that at symptomatic onset, motor unit numbers were severly reduced compared to wild type controls, with 68% loss in the hindlimb and 43% loss in the forelimb (Table 2). Relative to wild type controls, motor unit numbers were also severely reduced in clinically pre-symptomatic animals at the end of our study, with 81% loss in the hindlimb and 45% loss in the forelimb (Table 2).

**Table 2:** Electrophysiological deficits in clinically pre-symptomatic vs. symptomatic mutant SOD1 rats.

Gender	Longitudinal Matsumoto Motor Scores					MUNE at Onset of Motor Weakness (Motor Score = 4)	
	Baseline	5 days	10 days	15 days	20 days	Hindlimb	Forelimb
Female	5	4	3	—	—	214	273
Female	5	4	4	4	3	107	280
Male	5	5	5	5	4	139	107
Male	5	5	5	4	4	41	207
						**MUNE (% of Wild Type)**	
Mean Motor Unit Number in Wild Type Rats (n = 10; Mean ± Standard Deviation)						392 ± 65	384 ± 67
Mean Motor Unit Number in Clinically Pre-symptomatic Mutant SOD1 Rats at 20 Days (n = 5)						75 (19%)	212 (55%)
Mean Motor Unit Number in Mutant SOD1 Rats at Symptomatic Onset (n = 4)						125 (32%)	217 (57%)

4 of 9 mutant SOD1 rats (2 females and 2 males) demonstrated observable motor weakness during the course of our studies as determined by Matsumoto motor scoring. 1 female animal exhibited rapid functional decline and was removed from the study at the 10 day timepoint due to endpoint criteria while the remaining 3 clinically symptomatic animals displayed a more gradual decline in motor function. Mean motor unit number estimates of both the forelimb and hindlimb were significantly reduced in clinically symptomatic mutant SOD1 rats (n = 4) at motor weakness onset relative to wild type rats at baseline. The 5 remaining clinically pre-symptomatic animals, which did not display symptoms of motor weakness during the course of the study, exhibited profound reductions in forelimb and hindlimb estimated motor unit number values at 20 days, relative to wild type rats. Furthermore, hindlimb motor unit number estimation was lower at day 20 for clinically pre-symptomatic animals that never displayed motor weakness compared to hindlimb motor unit number estimation of clinically symptomatic animals at the time of motor weakness onset.

## Discussion

We reverse-translated the clinical MUNE technique for consistent application in the rat forelimb and hindlimb. Our standardised guidelines for placement of electrodes and acquisition of CMAP and SMUP waveforms resulted in robust and reliable forelimb and hindlimb recordings that can be leveraged for longitudinal preclinical assessments of MU integrity.

It is feasible to perform CMAP and MUNE recordings using both surface and needle electrodes. While preclinical electrophysiological studies often use needle recording electrodes^26,27^, we chose to use surface disc recording electrodes for several reasons. First, a primary goal of this study was to mimic clinical recordings as closely as possible, and clinical recordings are nearly universally performed using surface electrodes. Secondly, the unique potential of CMAP and MUNE is the ability to track motor unit integrity in the same animal over time. Therefore, a less invasive surface electrode approach was preferable. Lastly, our goal was to design a robust and comprehensive assay of the forelimb and hindlimb neuromusculature. Reliability of CMAP recordings are improved with larger electrode size^28^. Thus, a larger electrode surface was preferable over the smaller, more selective surface of a needle electrode. When more selective recordings are desired, needle recordings may be preferable, but it is also important to note that responses can vary greatly depending on electrode positioning (i.e. needle electrode depth and position in or around a muscle)^29,30^. Regardless of the type of recording electrode utilized, it is technically challenging to reliably record from individual muscles that are closely situated. Volume conduction from neighbouring muscles often contributes to recorded signals which limits selective recordings. Therefore, in our study we are not able to define the number of motor units in individual muscles of the forelimb and hindlimb. Instead, we recorded from regions overlying muscles with a common function and are hence able to define the number of motor units supplying distinct functional muscle groups (wrist flexors in the forelimb and ankle plantar flexors in the hindlimb). Importantly, in some conditions such as ALS and aging the susceptibility for motor unit degeneration may vary between muscles^31,32^. Therefore, continued work to develop methods for selective recordings from individual forelimb and hindlimb muscles would be beneficial.

For validation of our forelimb MUNE technique, we compared our measured MUNE values with summated motor neuron counts determined by retrograde labelling (Table 1). There was good agreement between anatomical motor neuron counts and electrophysiological estimation of MU number, supporting the validity of both techniques. The discrepancy between anatomical counts (336 ± 16 [SEM]) and electrophysiological estimates (415 ± 8 [SEM]) could be explained by the fact that the summated anatomical motor neuron counts do not include Flexor digitorum superficialis (FDS) muscle data, whereas the motor neurons supplying the FDS are included in forelimb MUNE values. Unfortunately, it is not possible to exclude the contribution of the FDS to the wrist flexor muscle group MUNE value for this comparison. Additionally, the variability in the numbers of motor neurons that are labelled between different animals could explain the discrepancy between anatomical counts and electrophysiological estimates. This is likely due to the fact that injecting each muscle requires spatial estimation of the motor end plate region, as observing this region *in situ* is currently not possible^33^. Variable uptake of FG at the neuromuscular junction likely results in partial labelling of muscle motor neuron columns and underestimation of the actual numbers innervating any given muscle^18,34^. However, we are confident that spurious labelling was not a result of tracer leakage, as applying FG to the external surface of a muscle’s intact fascia does not result in motor neuron labelling^34,35^. The quantification of FG-labelled motor neurons in a cohort of rats separate from the cohort that underwent MUNE analysis is a limitation of our approach. However, multiple muscles would need to be injected with retrograde tracer in order to compare motor neuron quantifications with MUNE in the same rat. Injecting all wrist flexor muscles in one rat forelimb would result in the FG-labelling of ~400–500 motor neurons, with the majority concentrating in the dorso-lateral aspect of the ventral horn between C6-C7 spinal cord. Due to the technical challenge of counting large numbers of motor neurons in this small region we decided that the most accurate way to determine the number of labelled motor neurons from multiple muscles was to inject the muscles individually, in separate rats, and summate the counts.

In the hindlimb, as the biceps femoris covers the majority of the motor end plate region of the gastrocnemius, and the soleus is located deeper, motor neuron counts using retrograde labelling with FG were not available for comparison with electrophysiological estimates. Mohan *et al*. (2015) did quantify the number of motor neurons innervating the gastrocnemius, but we believe that these values are a significant under-representation of the true number of innervating motor neurons since a majority of the motor end plate region of the gastrocnemius could not be targeted for FG injection. However, given the proximity of our anatomical and electrophysiological quantifications in the forelimb, we are confident that the MUNE results from the hindlimb are an appropriate and accurate estimate of triceps surae motor units. Further, MUNE has been previously established in the rat triceps surae muscle group, and our results (459 ± 10 [SEM]), using an analogous methodology to what we applied to the forelimb, were similar to rat triceps surae MUNE values in a previous report (385 ± 84 [SEM])^22^.

Electrophysiological measurements of CMAP and MUNE offer powerful potential as biomarkers in clinical and preclinical studies. Both CMAP and MUNE have been used as prognostic biomarkers showing strong correlation with disease severity, most notably in ALS and SMA^36,37^. In our studies we chose to use the incremental MUNE technique due to its simplicity and the requirement of only a single stimulation site. A variety of other MUNE techniques have been developed including multipoint stimulation^38^, combined multipoint incremental^13^, Bayesian statistical^39^, and motor unit number index (MUNIX)^40^. The Bayesian statistical MUNE method assesses variability of CMAP size at a variety of levels of submaximal stimulation to estimate SMUP sizes and assumes all-or-none responses of individual motor units. This statistical MUNE method has been shown to be problematic in ALS patients due to the loss of neuromuscular junction transmission fidelity and thus variability in individual motor unit responses between stimuli^41^. Further, in wild type mice Bayesian MUNE estimated only half of the motor neurons counted by histochemical analysis of the spinal cord^17^. Multiple point stimulation techniques rely on stimulation at different sites, requiring insertion of stimulation electrodes at multiple sites, and are more challenging in smaller animal models. Lastly, the MUNIX requires graded voluntary contractions of the muscle of interest which is not possible in sedated rats. Comparison between different MUNE methods in clinical studies has been hindered by a lack of a gold standard. Due to the aforementioned limitations we chose to compare our results from the incremental MUNE technique to labelled motor neuron counts instead of to other MUNE techniques. Future preclinical studies could be designed to compare labelled motor neuron counts with a variety of MUNE methods.

Despite the myriad advantages of an objective electrophysiological measure of motor unit loss, the limited application of MUNE in pre-clinical rodent studies has been restricted to the hindlimb^22,26,42,43^. The goal of this study was to develop and validate forelimb electrophysiological measures of motor unit connectivity as biomarkers of spinal motor neuron degeneration/dysfunction in the rat. Utilisation of forelimb and hindlimb CMAP and MUNE techniques in conjunction will allow for spatial characterisation of motor unit pathology in preclinical models of neuromuscular disease that were previously limited to hindlimb recordings. This represents an important advance, as many disorders can predominantly affect the upper or lower limbs in patients with different pathologies of the neuromuscular system. Spinal cord injury (SCI) research is one particular area where the ability to assess different spinal cord regions and pathologies (i.e., upper or lower motor neuron dysfunction) may be of particular benefit^44^. A clinical study found that CMAP amplitude was decreased in paralyzed muscles of all SCI patients, but that the amplitudes were more significantly decreased in SCI patients with little functional recovery compared to SCI patients with larger gains in functional recovery^45^. Since the majority of SCIs are the result of cervical insult^46^ the forelimb and hindlimb recording techniques presented here can be applied to experimental cervical SCI models to expand upon this important clinical finding and to understand the combined effects of upper and lower motor neuron dysfunction. Additionally, low replicability and robustness of behavioural assays of functional recovery limit the translation of findings in preclinical SCI models^47,48^. Therefore, objective measures of electrophysiological and physiological outcomes will optimise the predictive value of experimental SCI studies.

By applying our novel forelimb recording technique and the previously established hindlimb recording technique to an ALS rat model, we showed that electrophysiological measures can sensitively identify motor unit pathology in the forelimb, in addition to the hindlimb. Baseline measurements were timed according to previously described average age of onset of motor symptoms, and we timed our repeated measurements at 10 and 20 days after baseline since the average time to endpoint from the onset of clinical phenotype is 11 days^49,50^. At baseline, we found striking changes in both electrophysiological and muscle contractility profiles of the mutant rats despite the absence of obvious symptoms of motor weakness (clinically “pre-symptomatic”) using motor scoring^51^. These findings are aligned with prior longitudinal studies that have shown electrophysiological deficits (CMAP and MUNE) and muscle contractility deficits in the G93A mouse model of ALS prior to onset of clinical phenotype^43,52^. In the hindlimb, males had lower CMAP values and females exhibited higher SMUP values, irrespective of genotype. These findings are likely related to gender-specific anatomical differences in limb and muscle size. In contrast to the gender-specific differences for CMAP and SMUP, which represent the summated excitation of muscle or group of muscles being tested and the summated excitation of a single motor unit, respectively, the findings of similar MUNE values in male and female rats suggests that there are no differences in motor unit number between males and females which is similar to a previous study in humans^53^. We also found a rapid decline in electrophysiological measures after baseline over a period of 20 days in both clinically pre-symptomatic and symptomatic mutant SOD1 rats.

During our longitudinal study, 2 of the 4 female mutants and 2 of the 5 male mutants demonstrated clinically-observable signs of motor weakness (clinically “symptomatic”). Motor unit number at onset of clinical symptoms in SOD1 rats (as determined by a Motor Score of 4) were 68% reduced in the hindlimb and 43% reduced in the forelimb compared with wild type rats. These findings align well with prior reports of 50% spinal cord motor neuron count reduction at Motor Scores of 4^51^. Furthermore, we found a strong correlation between *in vivo* muscle contractility and CMAP and MUNE measures in the hindlimb. This provides physiological validation of the measure and supports the usefulness of electrophysiological assessments of lower motor neuron number and function. Interestingly, when we compared endpoint motor unit numbers from the clinically pre-symptomatic 5 mutant rats that exhibited no symptoms of motor weakness throughout the study to motor unit numbers from the wild type rats, we found 81% reduction in the hindlimb and 45% reduction in the forelimb. These findings are surprising, but the motor score does not directly measure muscle weakness and can be influenced by behavioral differences between animals such as variable effort due to increased motivation or apathy between animals as well as differing pathologies in different animals (i.e. burden of upper versus lower motor neuron loss). The CMAP, MUNE, and muscle contractility measures only assess lower motor neuron function. Therefore, the lack of congruency between symptom severity and CMAP, MUNE, and contractility is not surprising and could reflect heterogeneity of upper motor neuron involvement between the clinically symptomatic and pre-symptomatic groups of mutant rats or other behavioural differences between animals. In future studies, inclusion of outcomes that allow assessment of upper motor neuron connectivity such as motor evoked potentials could help clarify these findings.

There were several limitations of our study that may be addressed in future investigations. First, despite the well-established hindlimb contractility measurement there are no analogous methods available for assessing contractility in the forelimb of the rat. Forelimb grip is one measure that can assess muscle function, but grip (similar to the clinical score) can only assess the cumulative effects of upper and lower motor neuron dysfunction and thus suffers from limitations previously mentioned in the clinical motor score^54^. Invasive *in situ* contractility measuring techniques can be applied to the rat forelimb muscles but were not included as they are terminal experiments and are not suitable for longitudinal assessment^55^. Second, we chose to investigate the SOD1 rat model as a validation of our technique, but we did not define the full natural history of motor unit loss in this model. Similar to what we have performed in the G93A SOD1 mouse model^43^, it would be beneficial for future therapeutic development and testing to perform a more comprehensive study to understand when motor unit losses first occur in this model.

In conclusion, we have refined electrophysiological measures of motor unit connectivity in the rat forelimb and hindlimb. Scaling down clinical electrophysiological techniques for application in the rat engenders the discussed inherent challenges and limitations that future studies and technical advances will be necessary to address. However, we have successfully validated our techniques by performing test-retest reliability, anatomical motor neuron correlations, and testing these measures for sensitivity in a disease model. The use of both forelimb and hindlimb measures together can provide insight into topographic aspects of disease onset and progression in regard to regional loss of motor unit integrity. Furthermore, the repeatable *in vivo* applicability of CMAP and MUNE techniques allow longitudinal studies to characterise motor unit pathology and monitor the effects of therapeutic efforts. The ability of forelimb and hindlimb MUNE to objectively quantify MU integrity will facilitate clinically relevant advances in rat models of spinal cord and neuromuscular disease.

## Methods

### Animals

For the electrophysiological and muscle contractility experiments, male and female Sprague Dawley rats were group-housed (n = 2 per cage) with a standard 12-hour light and dark cycle at the animal facilities of The Ohio State University (Columbus, OH) and were provided continuous access to chow and water. A cohort of male Sprague Dawley rats (n = 6) was used to develop and establish the reproducibility of recording techniques. A separate cohort of rats hemizygous for the G93A transgene (5 mutant males and 4 mutant females) and wild type control rats (5 wild type males and 5 wild type females) on Sprague Dawley genetic background (Model number: 2148) were obtained from Taconic Biosciences^49,50^ for experiments in a disease model. For both studies, animals were assessed at 20–30 weeks of age, and all studies were carried out in accordance with and approved by The Ohio State University Wexner Medical Center’s Institutional Animal Care and Use Committee. The retrograde tracer experiments were previously conducted in a separate rat cohort^56^, but the data were reanalysed here.

### Experimental design and timeline

#### Experiment 1 – forelimb and hindlimb CMAP and MUNE recording techniques established in naïve rats

A single rater (M.E.H.) obtained forelimb and hindlimb recordings from six wild type male Sprague Dawley rats on three occasions (Days 1,3,5), with one rest day between recording sessions (Days 2,4). The rater was blinded to the prior CMAP and SMUP results, and MUNE values were not calculated until all data collection was completed.

#### Experiment 2 – comparison of motor neuron counts determined by anatomical labelling and electrophysiological estimation

MUNE values obtained from experiment 1 were compared to back-traced anatomical lower motor neurons from corresponding muscles in a historical cohort of naïve rats^56^.

#### Experiment 3 – characterisation of longitudinal motor unit and muscle contractility decline in SOD1 mutants

Prior phenotypic characterisation of the colony from which we obtained the animals used in the current study demonstrated a range of motor weakness onset between 161 and 217 days (Mean: 187 days)^49,50^. On this basis, we obtained baseline measures in SOD1 rats at 159–168 days of age (n = 4 mutant female SOD1 rats, mean age of 166 days [range: 161–168 days]; n = 5 male SOD1 rats, 159 days of age). Wild type females (n = 5, 168 days of age) and males (n = 5, 180 days of age) were used as controls. Phenotypic stage of disease was determined by behavioural Matsumoto motor scoring^51^. Baseline electrophysiology, muscle contractility, and Matusumoto Motor Score were performed by raters blinded to genotype (M.E.H. and W.D.A). Electrophysiological and muscle contractility measurements were repeated in the SOD1 mutants at 10 ± 1 and 20 ± 1 days after the baseline measurement.

### Anaesthesia and animal preparation

For electrophysiological and muscle contractility studies, anaesthesia was induced with 5% isoflurane (Piramal Healthcare, Mumbai, India) at 500 mL O_2_ flow per minute and maintained with 2% isoflurane at 300 mL O_2_ flow using a Somnosuite^®^ low-flow anaesthesia system (Kent Scientific, Torrington, CT). Anaesthesia maintenance was adjusted as necessary according to animal respiratory rate, and appropriate depth of anaesthesia was confirmed by lack of response to forceps application of light foot pinch. Body temperature (37 °C) was maintained by an infrared heating pad (provided with anaesthesia system) to avoid temperature-dependent changes in CMAP, and Puralube vet ointment (Dechra, Northwich, UK) was applied to prevent ocular dryness. Fur was completely removed from the left forelimb and right hindlimb with shaving clippers to ensure consistent placement of stimulating electrodes and optimal measurements from recording electrodes.

### *In vivo* electrophysiology

For forelimb electrophysiological measurements, animals were placed in the supine position and forelimbs affixed with Transpore tape (3 M, Maplewood, MN). E1 (active) and E2 (reference) TECA 6030-TP surface disc recording electrodes (Natus Neurology, Middleton, WI) were affixed directly to the ventromedial forearm overlying the bulk of the left wrist flexor muscles (flexor carpi radialis m. [FCR], palmaris longus m. [PL], flexor digitorum profundus m. [FDP], flexor digitorum superficialis m. [FDS]) and left ventral carpus/metacarpus, respectively. Prior to adhesion to the skin, the recording electrodes were coated with Spectra 360 electrode gel (Parker Labs, Fairfield, NJ) to minimise skin-electrode electrical impedance. A disposable tab adhesive electrode (Natus Neurology, Middleton, WI) was used as a ground electrode and attached to the tail. TECA elite disposable insulated monopolar 28 G needles (Natus Neurology, Middleton, WI) were used as stimulating anode and cathode electrodes. Both stimulating electrodes were placed 2 cm from the midline with the anode inserted subcutaneously, just rostral to the left pectoralis muscle, and the cathode caudally inserted subcutaneously over the mid-left pectoralis muscle. The distance between the anode and cathode was 1 cm (see Fig. 2A).

Hindlimb electrophysiological measurements followed a similar set-up except animals were positioned supine, and active and reference surface disc recording electrodes were affixed over the bulk of the right triceps surae (medial gastrocnemius m., lateral gastrocnemius m., soleus m.) and Achilles tendon, respectively. Both stimulating electrodes were placed 3 cm from the midline with the anode inserted subcutaneously just caudal to the right femur and the cathode inserted subcutaneously in the mid-thigh. The distance between the anode and cathode was maintained at 1.25 cm (see Fig. 3A).

CMAP and SMUP measurements were obtained, as described previously, with clinical electrodiagnostic systems (Synergy EMG machine version 9.1, Oxford Instruments, Abingdon, UK; Cadwell Sierra Summit, Kennewick, WA)^57^. Brachial plexus or sciatic nerve were stimulated with square wave pulses for a duration of 0.1 ms at a frequency of 1 Hz, and low and high frequency filter vales were maintained at 10 Hz and 10 kHz, respectively. Recording sessions extended no longer than 20–30 minutes per animal.

CMAP responses were measured by stimulating the left brachial plexus (forelimb) or right sciatic nerve (hindlimb) with increasing intensity from 1–20 mA with a duration of 0.1–0.2 ms until a maximum response was achieved. To confirm the maximal CMAP response, a supramaximal stimulation (120% of previous stimulus intensity) was applied and lack of an additional increase in CMAP amplitude confirmed. Measurements were obtained at a constant screen sensitivity and duration setting (Sensitivity: 200 mV, 20 mV per division; Duration: 10 ms, 1 ms per division), and both baseline-to-peak and peak-to-peak CMAP values were recorded in mV.

An incremental technique was applied to determine average SMUP responses in the wrist flexor and triceps surae muscle groups^7,57,58^. Stimulations, starting at an intensity of 0.03 mA, were continuously applied at a frequency of 1 Hz to the left brachial plexus (forelimb) or right sciatic nerve (hindlimb). Current intensity was increased by 0.03 mA steps until the first all-or-none SMUP response was obtained. Once the initial response was obtained, the second SMUP was obtained by further increasing the intensity in 0.03 mA increments until a larger all-or-none response was evoked. This process was repeated to acquire a total of 10 SMUP responses. To facilitate consistent response collection and to avoid fractionation and alternation of incremental responses, responses were superimposed and acquired in real-time. Each incremental response was selected only if it aligned temporally with the supramaximal CMAP response, was observed three times, and was at least 50 µV larger than the previous SMUP response. Measurements were obtained with a constant screen sensitivity and duration setting (sensitivity: 2 mV, 200 µV per division; duration: 10 ms, 1 ms per division). Incremental responses were recorded in µV, and average peak-to-peak SMUP values were determined by dividing the 10^th^ and final SMUP by ten, the total number of increments. Average peak-to-peak SMUP values were divided into corresponding peak-to-peak CMAP values to estimate the number of functional MUs innervating the wrist flexor or triceps surae muscle groups.

### Fluoro-gold labelling of the rat wrist flexor muscles

Retrograde tracer experiments characterising the spatial distribution of forelimb-supplying motor neuron columns for a vast array of muscles were previously performed and published in a 2012 study by Tosolini and Morris; however, the quantifications are presented for the first time here. Briefly, for each muscle, FG-labelled motor neurons were represented on a spinal cord diagrammatic schematic as a single dot, but the number of innervating motor neurons was not quantified. We reanalysed these data to determine anatomical motor neuron counts for the wrist flexor muscles of interest: FCR, PL, and FDP (data for FDS not available).

### Muscle contractility

Plantar flexion muscle contractility was assessed in SOD1 mutant rats and naïve controls using an *in vivo* muscle contractility apparatus (Model 1305 A, Aurora Scientific Inc, Canada) similar to what we have previously described in mouse models of aging and ALS^42,43^. The paw of the right hindlimb was taped to a force plate and the tibia and foot were aligned at 90°. The knee was secured with a blunt clamp at the femoral condyles. A pair of disposable monopolar electrodes (Natus Neurology Inc, Middleton, WI, USA) were placed subcutaneously in the region of the medial posterior leg in the region of the tibial branch of the sciatic nerve. Maximum twitch torque was measured following a single supramaximal stimulation (0.20 ms square wave pulse). Subsequently, maximum tetanic muscle contraction was assessed by stimulating the tibial nerve with a 500 ms train of stimuli at a stimulation rate of 125 Hz. Absolute twitch and tetanic torque values normalised to body mass were used for comparisons.

### Matsumoto motor score

A 5-point Matsumoto Motor score, as described previously^51^, was obtained at baseline and every 5 (±2) days thereafter for mutant SOD1 rats. In brief, the animals were objectively scored for the ability to right themselves from their sides and for the ability to stand up on their hindlimbs. A rat with no motor impairment (clinically pre-symptomatic) received a score of 5, while a rat with a score of 0 was barely able to move voluntarily.

### Statistics

Statistical analyses were performed using GraphPad Prism 8 software (GraphPad Software, Inc., San Diego, CA). Data from experiments 1 and 2 (Figs 2, 3; Table 1) are presented as mean with standard error of the mean, and data from experiment 3 (Figs 5, 6; Table 2) are presented as mean with standard deviation. To assess consistency between repeated measures in the naïve rat forelimb and hindlimb for CMAP, SMUP, and MUNE, coefficients of variation were calculated between repeated measures by comparing absolute % difference versus mean. Two-way ANOVA was used for comparison of grouped data obtained from baseline measurements in wild type and SOD1 male and female mutant rats. One-way ANOVA with Dunnett’s multiple comparisons test was used to assess for longitudinal decline of electrophysiological and muscle contractility parameters in the male and female mutant SOD1 rats. Linear correlations were determined with Pearson correlation coefficient. A p-value of <0.05 was considered significant.

## Data Availability

The datasets generated during these studies are available from the corresponding author upon request.
